# Effects of Water Source, Sanitation and Hygiene on the Prevalence of *Schistosoma mansoni* among School Age Children in Jawe District, Northwest Ethiopia

**Published:** 2020

**Authors:** Tadesse HAILU, Wondemagegn MULU, Bayeh ABERA

**Affiliations:** Department of Microbiology, Immunology and Parasitology, College of Medicine and Health Science, Bahir Dar University, Bahir Dar, Ethiopia

**Keywords:** *Schistosoma mansoni*, Sanitation, Hygiene, Pure water

## Abstract

**Background:**

Intestinal schistosomiasis is a disease caused by infection with one of the blood flukes called *Schistosoma mansoni. The distribution of Schistosoma mansoni* infection is high in Sub-Saharan Africa due to water source, sanitation and hygiene problems. This study aimed to determine the effect of water source, sanitation and hygiene on the prevalence of schistosomiasis among school-age children in Northwest Ethiopia.

**Methods:**

A cross-sectional study was conducted from Apr 2016 to Aug 2016. Children were selected by systematic random sampling and Formol Ether Concentration Technique (FECT) was used to identify *Schistosoma mansoni* infection. Statistical analysis was done using descriptive statistics and strength of association of schistosomiasis with determinant factors was calculated by bivariate analysis.

**Results:**

Among 333 children, 7% were infected with *Schistosoma mansoni.* Using surface water for drinking, poor hand wash habit and latrine utilization were significantly associated (*P*<0.05) with *Schistosoma mansoni* infection.

**Conclusion:**

Absence of safe water for bathing, washing and swimming, poor sanitation and hygiene practices were major risk factors for schistosomiasis. Therefore, health education should be given on the transmission of *S. mansoni* infection, pure water, sanitation and hygiene in *S. mansoni* endemic areas.

## Introduction

Schistosomiasis is water based parasitic infection that affects more than 800 million people globally, and more than 90% live in sub-Saharan African countries with poor access to clean water and sanitary facilities (1). WHO estimates that there are about 20,000 deaths of schistosomiasis globally each year *(2). Schistosoma (S) mansoni* is one of the causes of intestinal schistosomiasis (3,4).

Transmission of *S. mansoni* is through exposure of skin to faecal contaminated freshwater containing cercarial stages. The eggs emerge larvae when contact with water and enter freshwater snails for further development. Finally, the cercarias come out from snail and penetrate the human skin during contact with infested water (5).

The prevalence of intestinal schistosomiasis is influenced by Water, Sanitation, Hygiene (WASH) and mass drug administration (6). Contact with freshwater during washing cloth, bathing and crossing is the main risk factor (7). Open defecation and poor hand wash habit are indicators of WASH and influence the prevalence of schistosomiasis (5).

Access to safe water and adequate sanitation are considered to be important components of schistosomiasis control, which at present largely relies on preventive chemotherapy with a single drug, praziquantel (8). Even though re-infection may occur after treatment, the risk of developing severe disease is diminished and even reversed (2). Treatment alone will not break the cycle of transmission; improvements of WASH, infrastructure and appropriate health-seeking behavior are essential to achieve sustained control of schistosomiasis (9). Therefore, integration of WASH and mass drug administration might increase preventive capacity to a higher rank.

School-age children usually being the most affected group since they are playing with water (10). In low socio-demographic areas, there is poor water supply, latrine utilization; sanitation and hand wash habit which facilitates the *S. mansoni* transmission. Institutional based information indicated that the prevalence of *S. mansoni* is one of the primary helminthic infections among children in Jawe district. However, the available information with respect to WASH and *S. mansoni* infection is lacking. Therefore, this study aimed to determine the effect of WASH on *S. mansoni* infection among school age children, Northwest Ethiopia.

## Methods

### Study design, area and period

This cross sectional study was conducted among febrile school age children from Apr 2016 to Aug 2016 in Jawe district, Northwest Ethiopia. The annual temperature of the area ranges between 16.68 °C to 37.6 °C. The average annual rainfall is 1569.4 mm.

Overall, 333 school age children were included in this study. Systematic random sampling technique was conducted until the required sample size is achieved. The samples were collected in Jawe health center and Workmeda health center.

All children age ranging from 6–14 yr, attending the above health centers and willing to participate in the study were included. Children under taking anti-helminthic drugs during the data collection time were excluded. The sample size in each health center was allocated by considering the population in the catchment areas. Jawe district is a potential irrigation area to sugar cane using Tana-Beles irrigation project.

### Data collection

Demographic information, indicators of WASH and environmental related factors were collected via interview of parents/guardian of the children by health officers.

Informed consent was taken from the participants before the study.

### Stool sample collection

Fresh stool specimen was collected from each study participant using clean plastic container labeled with unique identification number. In Formol Ether Concentration Technique (FECT), 0.5 g of stool sample was transferred in to 10 ml of normal saline in a glass container and mixed thoroughly. Two layers of gauze were placed in a funnel and strained the contents into a 15 ml centrifuge tube. Then 2.5 ml of 10% formaldehyde and 1 ml of ether was added. The test tubes were mixed well and centrifuged at 1000 revolution for three minutes. The sediment was mixed well, prepared on slide and covered with cover slide and saw with microscope.

### Quality control

Training of laboratory technicians and health officers on data collection was given before sample collection. Application of standard procedures was checked. The stool cups were labeled based on their serial number. The FECT slides were examined independently with two experienced laboratory technicians and 10% of FECT slides was randomly selected and read by another technician as a quality control. The results of their observation were recorded for later comparison on separate sheets.

### Data Analysis

Data were analyzed using SPSS ver. 20 (Chicago, IL, USA) statistical software. Overall magnitude of *S. mansoni* was calculated using descriptive statistics and chi-square. Strength of association between *S. mansoni* infection and indicators of WASH was calculated by logistic regression and calculating the odds ratios with 95% CI. The differences were considered to be statistically significant if *P*-value < 0.05.

## Results

### Demographic characteristics

Overall, 333 school age children included in the study; of which 275 (82.6%) from rural with response rate of 94.5%. Female participants accounted for 51.1%. The median age of children was 12 yr with standard deviation of 2.7. The majority of participants were Christian (98.4%) (Table 1).

**Table 1:** Demographic characteristics of school age children in Northwest Ethiopia, 2016 [N, %]

***Variables***		***N***	***S. mansoni distribution***		**P *value***
			***Positive***	***Negative***	
Age(yr)	6–10	143	6 (4.2)	137 (95.8)	0.10
	11–14	190	17 (8.9)	173 (91.1)	
Sex	Male	163	16 (5.5)	154 (94.5)	0.07
	Female	170	7 (8.2)	156 (91.8)	
Religion	Christian	328	23 (7.6)	305 (92.4)	0.54
	Muslim	5	0 (0)	5 (100)	
Residence	Rural	273	20 (7.3)	253 (92.7)	0.52
	Urban	60	3 (5)	57 (95)	
Total		333	23 (7)	310 (93)	

### Schistosoma mansoni infection

The overall prevalence of *S. mansoni* infection among school-age children was 23 (7%). The prevalence of *S. mansoni* among children age groups 11–14 and 6–10 were 8.9% and 4.2%, respectively. The prevalence of *S. mansoni* infection among rural dwellers was 7.3% (Table 1).

### Multivariate analysis of hookworm infection

Children with stream water source were 13.66 (AOR) times more likely to be infected by *S. mansoni* than who had pipe water source. Children bathing, fishing, swimming, crossing and washing cloth in surface water were 20.24 (AOR), 7.56 (AOR), 24.04 (AOR), 8.68 (AOR), and 8.99 (AOR) times more likely to be infected by *S. mansoni*, respectively. Children washed their hands sometimes were 12.25 (AOR) times more likely to be infected by *S. mansoni* than who washed their hands always. Children who used latrine sometimes were 7.99 (AOR) times more to be infected by *S. mansoni* than who used latrine always (Table 2).

**Table 2:** Determinant factors of *S. mansoni* among school age children in Northwest Ethiopia, 2016

***Variable***		***PI***		***AOR [95%CI]***	**P*-value***
		***Infected***	***Non infected***		
Water source	Surface	19	82	13.66 (1.42–131.91)	0.02
	Pipe	4	228		
Bathing in surface water	Yes	18	87	20.24 (1.29–16.74)	0.03
	No	5	223		
Fishing in surface water	Yes	15	37	7.56 (1.30–44.19)	0.02
	No	8	273		
Swim in surface water	Yes	18	65	24.04 (2.33–247.63)	0.01
	No	5	245		
Crossing in surface water	Yes	19	75	8.68 (1.24–60.51)	0.03
	No	4	235		
Washing clothes in surface	Yes	18	57	8.99 (1.50–53.95)	0 .02
water	No	5	253		
Hand wash habit	Sometimes	20	128	12.25 (1.46–102.86)	0.02
	Always	3	182		
Latrine utilization	Sometimes	16	64	7.99 (1.36–46.80)	0.02
	Always	7	246		

## Discussion

Water, sanitation and hygiene are crucial for prevention and control of Neglected Tropical Diseases including schistosomiasis. The prevalence of schistosomiasis is highly influenced by WASH and mass drug administration (5).

The prevalence of *S. mansoni* in the present study was 7% among school-age children, which was comparable with previous study done in Northeastern Nigeria (11), but lower than a study done in Northwest Ethiopia (12), Adwa, Northwest Ethiopia (13) and Southwest Ethiopia (14) and higher than a study done in Gondar town, Northwest Ethiopia (15), and Mali, West Africa (16). The difference might be due to the difference in geographical area, Socio-demographic, methodology, WASH, control strategies and annual de-worming.

In the present study, high prevalence of *S. mansoni* was found among children with age range 11–14 which was comparable with previous study done in Adwa, Northwest Ethiopia (13) and Southwestern Nigeria (17).

In the present study utilization of surface water source for home activities is a determinant factor for schistosomiasis which was in agreement with Southwest Ethiopia (14) and Southwestern Nigeria (17).

In our study, fishing in surface water was risk factor for children to be infected with *S. mansoni*. This result was in line with previous study done in Northeastern Nigeria (13).

In this study, swimming in surface water was risk factor for children to be infected with *S. mansoni*. This result was in line with previous study done in Amibera District, Southern Ethiopia (18), and Northeastern Nigeria (11),

We found that bathing and washing clothes in surface water were risk factor for children to be infected with *S. mansoni*. This result was in line with previous study done in Adwa, Northwest Ethiopia (13).

Poor hand wash habits and latrine utilization are important factors for the high prevalence of helminthic infections (19). Hand washing habits and latrine utilization of children were associated with *S, mansoni* infection in the present study. Similar findings were recorded in Southwest Ethiopia (14), Nothwest Ethiopia (13).

## Conclusion

Absence of safe water for bathing, washing and swimming, poor sanitation and hygiene practices were major risk factors for schistosomiasis. WASH was not integrated with mass drug administration to prevent *S. mansoni* infection. Therefore, health education should be given on transmission of *S. mansoni* infection and WASH in endemic areas.

## References

[1] World Health Organization Working to overcome the global impact of neglected tropical diseases. WHO, Geneba, Switzerland, 2010 https://www.who.int/neglected_diseases/resources/9789241564090/en/

[2] WHO Schistosomiasis. Fact sheet February 2016. https://www.who.int/news-room/fact-sheets/detail/schistosomiasis

[3] Colley DG Bustinduy AL Secor WE King CH Humans schistosomiasis. Lancet. 2014; 383:2253–64 24698483 10.1016/S0140-6736(13)61949-2 PMC4672382

[4] World Health Organization (WHO) Schistosomiasis: population requiring preventive chemotherapy and number of people treated in 2010. Wkly Epidemiol Rec. 2012; 87(4):37–44. 22308580

[5] Grimes JET Croll D Harrison WE The roles of water, sanitation and hygiene in reducing schistosomiasis: a review. Parasit Vectors. 2015; 8: 156. 25884172 10.1186/s13071-015-0766-9 PMC4377019

[6] Johnston EA Teague J Graham JP Challenges and opportunities associated with neglected tropical disease and water, sanitation and hygiene intersectoral integration programs. BMC Public Health, 2015; 15:547 26062691 10.1186/s12889-015-1838-7 PMC4464235

[7] Akogun OB Water demand and schistosomiasis among the Guam people of Bauchi state, Nigeria. Trans R Soc Trop Med Hyg, 1990; 84:548–550. 2128667 10.1016/0035-9203(90)90034-c

[8] WHO Schistosomiasis: progress report 2001– 2011, strategic plan 2012–2020. Geneva: World Health Organization, 2013.

[9] Freeman MC Ogden S Jacobson J Integration of Water, Sanitation, and Hygiene for the Prevention and Control of Neglected Tropical Diseases: A Rationale for Inter-Sectoral Collaboration. PLoS Negl Trop Dis 2013; 7(9): e2439 24086781 10.1371/journal.pntd.0002439 PMC3784463

[10] Saathoff E Magnnssen PA Kvalsuing JC Patterns of *Schistosoma haemotobium* infection impact of praziquantel treatment in a cohort of school children from rural Kwazulu natal South Africa. BMC Infection Disease, 2004; 174: 1331–1355. 10.1186/1471-2334-4-40 PMC524490 15471549

[11] Houmsou RS Panda SM Elkanah SC Cross-sectional study and spatial distribution of schistosomiasis among children in Northeastern Nigeria. Asian Pac J Trop Biomed 2016; 6(6): 477–484

[12] Worku L Damte D Endris M *Schistosoma mansoni* Infection and Associated Determinant Factors among School Children in Sanja Town, Northwest Ethiopia. J Parasitol Res 2014; 2014: 792536. 24800058 10.1155/2014/792536 PMC3995176

[13] Legesse L Erko B Hailu A Current status of intestinal Schistosomiasis and soil transmitted helminthiasis among primary school children in Adwa Town, Northern Ethiopia. Ethiop J Health Dev, 2010; 24(3):191–197.

[14] Jejaw A Zemene E Alemu Y High prevalence of *Schistosoma mansoni* and other intestinal parasites among elementary school children in Southwest Ethiopia: a cross-sectional study. BMC Public Health, 2015; 15: 600. 26135566 10.1186/s12889-015-1952-6 PMC4488975

[15] Gelaw A Anagaw B Nigussie B Prevalence of intestinal parasitic infections and risk factors among schoolchildren at the University of Gondar Community School, Northwest Ethiopia: a cross-sectional study. BMC Public Health, 2013; 13:304 23560704 10.1186/1471-2458-13-304 PMC3621079

[16] Dabo A Diarra AZ Machault V Urban schistosomiasis and associated determinant factors among school children in Bamako, Mali, West Africa. Infect Dis Poverty, 2015; 4:4. 25973199 10.1186/2049-9957-4-4 PMC4429506

[17] Risikat SA Adegbite Ayoade AA Correlation analysis between the prevalence of *Schistosoma* *haematobuim* and water conditions: A Case Study among the School Pupils in Southwestern Nigeria. IJRRAS, 2012; 13 (1): 1–17.

[18] Awoke W Bedimo M Tarekegn M Prevalence of schistosomiasis and associated factors among students attending at elementary schools in Amibera District, Ethiopia. Open Journal of Preventive Medicine, 2013; 3: 2.

[19] Magalhãesa RJS Barnettb AG Clementsa ACA Geographical analysis of the role of water supply and sanitation in the risk of helminth infections of children in West Africa. PNAS, 2011; 108: 50. 10.1073/pnas.1106784108 PMC3250163 22123948

